# Mckusick-Kaufman Syndrome Presenting as Acute Intestinal Obstruction

**Published:** 2013-01-01

**Authors:** Ramesh B Hatti, Ashok V Badakali, R N Vanaki, Mahantesh S Samalad

**Affiliations:** Department of Pediatric Surgery, S.N.M.C and H.S.K hospital, Navanagar Karnataka, India; 1 Department of Pediatrics, S.N.M.C and H.S.K hospital, Navanagar Karnataka, India

**Keywords:** Hydrometrocolpos, McKusick-Kaufman syndrome, Neonatal intestinal obstruction

## Abstract

Hydrometrocolpos and polydactyly have been associated with many syndromes and can present at any age. Rarely does hydrometrocolpos present as neonatal intestinal obstruction. We report two cases of McKusick-Kaufman syndrome presenting with intestinal obstruction. In both cases, intestinal obstruction got relieved after a cutaneous vaginostomy.

## INTRODUCTION

Hydrometrocolpos (HMC) is a pathological distension of uterus and vagina with excessive amount of fluid, in presence of distal vaginal out flow obstruction. HMC alone is often seen in cloaca, imperforate hymen, urogenital sinus, transverse vaginal septum etc. McKusick-Kaufman syndrome (MKKS), Bardet-Biedl syndrome (BBS), Pallister-Hall syndrome, Ellis-Van Creveld syndrome and Orofaciodigital syndrome type IV are few syndromes that need to be considered when hydrometrocolpos is associated with postaxial polydactyly (PAP) [1]. MKKS was initially described by McKusick in the Amish population and less than 100 cases have been reported in English literature to date [2]. For females without a family history in non-Amish population, HMC with vaginal agenesis, PAP and a cardiac anomaly are considered sufficient clinical evidence of MKKS.

## CASE SERIES

**Case 1:** A 4-day-old, term female baby presented with severe abdominal distension and bilious vomiting. Antenatal ultrasound done elsewhere was suggestive of hydronephrosis with cyst in pelvis. Clinical examination revealed a markedly distended abdomen with prominent superficial veins and a palpable cystic mass occupying almost whole of the abdomen (Fig. 1). Peristalses were seen in the upper abdomen. There was normal anal opening with urogenital sinus and associated postaxial polydactyly in the all limbs. Ophthalmologic and cardiovascular system examinations were normal.

**Figure:**
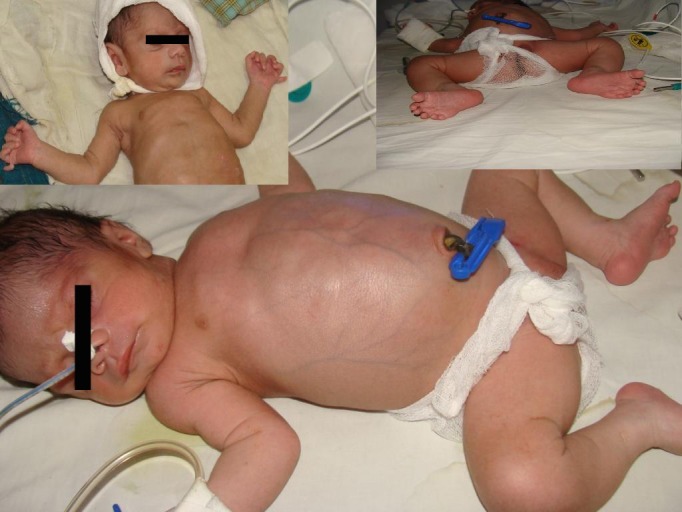
Figure 1: Photograph showing abdominal distension with polydactyly in all limbs.

**Figure:**
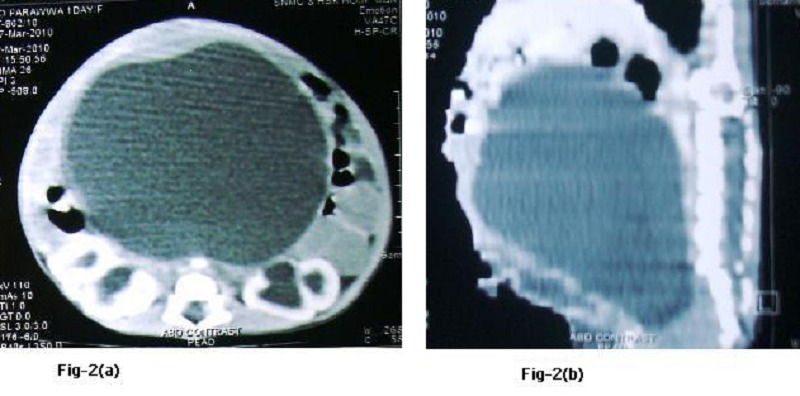
Figure 2 (a, b): CT images showing severe hydrometrocolpos.

A large abdominal mass displacing the bowel to upper abdomen was seen on plain abdominal radiograph. Ultrasound and CT scan findings (Fig. 2a and 2b) were consistent with the huge HMC compressing rectum and bladder. There was associated bilateral hydroureteronephrosis on CT scan. There was no communication between the bladder and vagina on genitogram (Fig. 3), which was done postoperatively. Neonatal cystoscope was not available at our centre to establish this finding. Echocardiography showed ASD. A cutaneous vaginostomy was created and Foleys catheter left in situ for 10 days. The neonate had an uneventful recovery and from postoperative day 1 baby started passing stools. Child is now thriving well and is 1 year old. She has been planned for definitive surgery.

**Case 2:** A 3-day-old female baby was admitted with bilious vomiting and abdominal distension. It was a term normal delivery and antenatal USG was not done. On examination baby had distended abdomen with palpable large cystic mass in lower abdomen. There was a normal anal opening with urogenital sinus and associated postaxial polydactyly in the all limbs. Ophthalmologic and cardiovascular system examinations were normal. 

USG suggested a large hydrometrocolpos. MRI scan (Fig. 4), showed large hydrometrocolpos with atretic lower vagina and associated bilateral hydronephrosis. Genitogram revealed no communication between bladder and vagina. Echocardiography suggested a VSD with a PDA. A cutaneous vaginostomy was done and post-operative recovery was uneventful. At 4 month follow up, child is thriving well and is awaiting definitive vaginal reconstruction.

**Figure:**
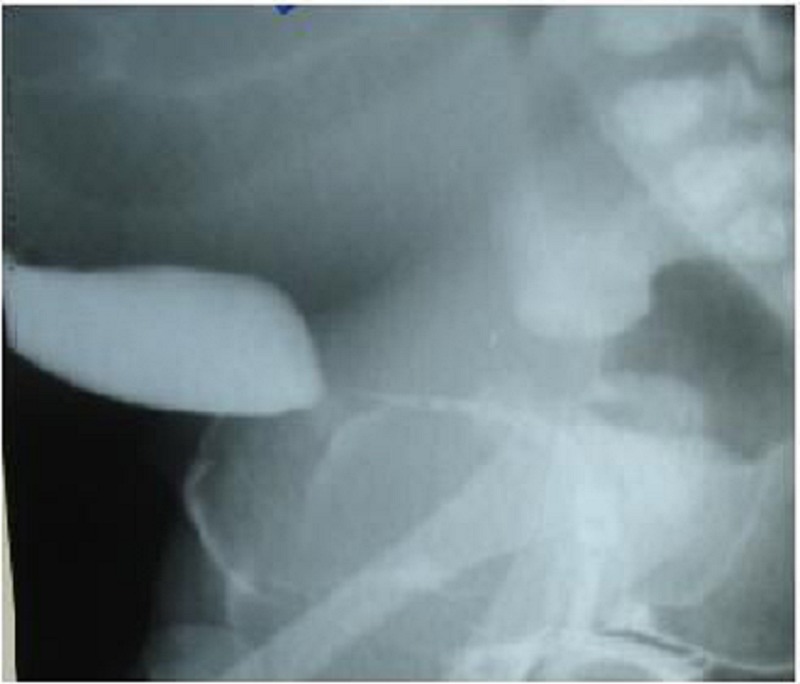
Figure 3: Genitogram shows no communication with vagina and urinary system.

**Figure:**
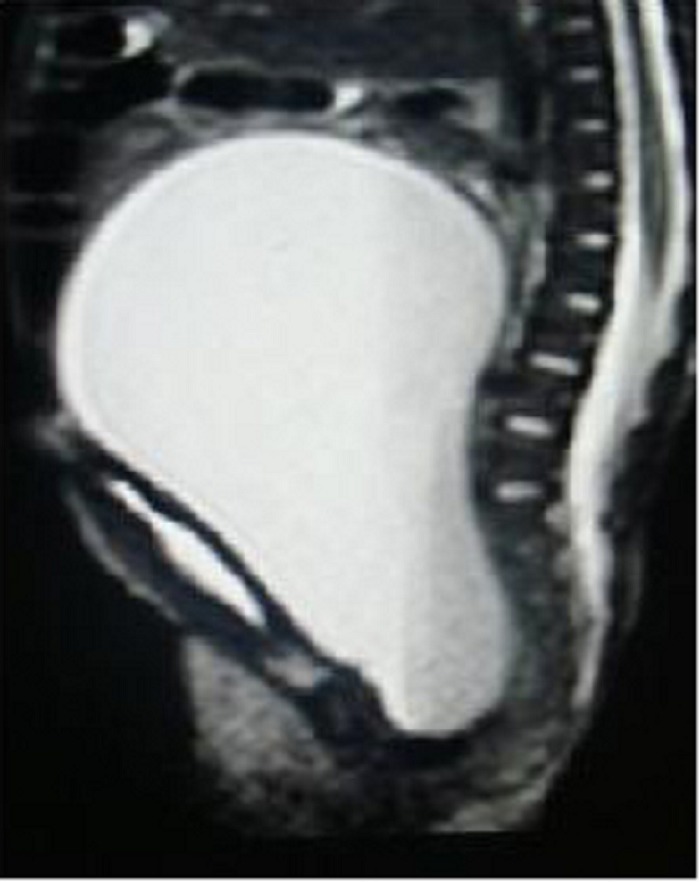
Figure 4: MRI of hydrometrocolpos with vaginal atresia.

## DISCUSSION

The presentation of hydrometrocolpos depends on the degree of compression by the enlarged uterus and vagina. Hydronephrosis is usually associated due to compression over bladder neck. Huge HMC can also cause bowel obstruction [3]. Both our cases presented as acute neonatal intestinal obstruction. A simple clinical examination including thorough perineal and rectal examination remains the main stay in diagnosis. Ultrasonography remains the preferred modality for the initial study. MR imaging or CT scan is suggested for patients with a technically inconclusive ultrasound examination and to find other uterine anomalies [4]. 

McKusick–Kaufman syndrome (MKKS) is a recessively inherited human genetic disease predominantly characterized by developmental anomalies, including vaginal atresia with hydrometrocolpos, polydactyly, and congenital heart defects [5]. Bardet-Biedl syndrome (BBS) is the generic name for a genetically heterogeneous group of autosomal recessive disorders characterized by retinitis pigmentosa (RP) or retinal dystrophy (appearing usually between 10 and 20 years of age), HMC, postaxial polydactyly, obesity, nephropathy, and mental disturbances, or, occasionally, mental retardation [1]. There are no phenotypic features that allow reliable differentiation between the two syndromes in the neonatal period [6]. Clinical features that allow discrimination between the two syndromes such as, features of retinitis pigmentosa, obesity, learning disability in BBS are age dependent [1, 6]. However, uterine, ovarian, and fallopian tube anomalies are more common in BBS patients. Heart defect has often been considered as a useful clue to the diagnosis of MKKS, but is of uncertain use in the differential diagnosis of BBS and MKKS. Hence sporadic female infants with HMC and PAP cannot be diagnosed as MKKS until at least age of 5 years [6]. Temporarily the vagina can be usually drained by a catheter introduced via the urogenital sinus or else a cutaneous vaginostomy should be performed [7, 8]. Both of our babies were relieved of intestinal obstruction after a cutaneous vaginostomy and are planned for a definitive vaginal reconstruction.

## Footnotes

**Source of Support:** Nil

**Conflict of Interest:** None
